# Entropy-Based Applications in Economics, Finance, and Management

**DOI:** 10.3390/e24101468

**Published:** 2022-10-14

**Authors:** Joanna Olbryś

**Affiliations:** Faculty of Computer Science, Bialystok University of Technology, Wiejska 45a, 15-351 Białystok, Poland; j.olbrys@pb.edu.pl

The concept of entropy originated from physics (precisely, from thermodynamics), but it has been utilized in many research fields to characterize the complexity of a system and to investigate the information content of a probability distribution. Entropy is a general measure, and therefore, many definitions and applications of entropy have been proposed in the literature.

This Special Issue of *Entropy* was intended to be a forum for the presentation of entropy-based applications in economics, finance, and management studies. The thirteen high-quality articles included in this Special Issue propose and discuss new tools and concepts derived from information theory to investigate various aspects of entropy with assorted applications.

In the first contribution [1], the authors propose a market clustering measure using granular trading data and the maximum-entropy concept. The effect of crowded trades on stock price stability is investigated, and the evidence is that market clustering has a causal effect on the properties of the tails of the stock return distribution, particularly the positive tail, even after controlling for commonly considered risk drivers. Reduced investor pool diversity could thus negatively affect stock price stability.

The second paper [2] introduces a new methodology for the measurement of stock market depth and market liquidity. The proposed Shannon entropy-based market depth indicator is supported by an algorithm inferring the initiator of a trade. The findings of empirical experiments for real high-frequency data indicate that this new entropy-based approach can be considered as an auspicious market depth and liquidity proxy with an intuitive base for both theoretical and empirical analyses in financial markets.

The aim of the third contribution [3] is to conduct a dynamic analysis based on generalized vector autoregressive volatility spillover variance decomposition, construct a complex network, and adopt the minimum spanning tree method to clarify and analyze the risk propagation path between different bond types in China’s bond market. The network’s structural entropy is calculated as a useful indicator of the complexity of the network system.

The goal of the fourth paper [4] is to identify the degree of coherence of credit cycles in the countries potentially seeking to adopt the euro with the credit cycle inside the Eurozone. The indicators that define the credit cycle similarity and synchronicity in the selected countries and a set of entropy-based measures (i.e., the block entropy, the entropy rate, the Bayesian entropy) are calculated.

In the fifth paper [5], the authors try to establish the commonalities and leadership in the cryptocurrency markets by examining the mutual information and lead–lag relationships between Bitcoin and other cryptocurrencies. The transfer entropy between the volatility and liquidity of seven highly capitalized cryptocurrencies is calculated in order to determine the potential direction of information flow. Empirical results suggest the gradual increase in the role of privacy-oriented cryptocurrencies.

The sixth contribution [6] presents an extension of the Technique for Order Preference by Similarity to Ideal Solution (TOPSIS) method with objective criteria weights for Group Decision Making (GDM) with Interval Numbers (INs). The proposed method is an alternative to popular and often used methods that aggregate the decision matrices provided by the decision makers (DMs) into a single group matrix, which is the basis for determining objective criteria weights and ranking the alternatives. The objective criteria weights are calculated using the interval entropy method. The numerical example shows the ease of use of the proposed method, which can be implemented in common data analysis software.

The seventh paper [7] analyzes the changes in the financial network built using the Dow Jones Industrial Average components following monetary policy shocks. Monetary policy shocks are measured through unexpected changes in the federal funds rate in the United States. The changes in the financial networks using singular value decomposition entropy and von Neumann entropy are investigated. The results indicate that unexpected positive shocks in monetary policy shocks lead to lower entropy.

In the eight paper [8], the main research question concerns the identification of changes in the COVID-19 epidemiological situation using fuzzy clustering methods. The identification of country types in terms of epidemiological risk is carried out using the fuzzy c-means clustering method. Moreover, the entropy index is used to measure the degree of fuzziness in the classification and evaluate the uncertainty of epidemiological states. The research concerns Europe, but the methodology is universal and can also be useful for other countries.

The purpose of the ninth contribution [9] is to compare the risk transfer structure in Central and Eastern European and Western European stock markets during the 2007–2009 financial crisis and the COVID-19 pandemic. A variety of methods, including mutual information and transfer entropy, are used. The results indicate that there are significant nonlinear correlations in the capital markets that can be practically applied for investment portfolio optimization. The study provides an insight into the risk transfer theory in developed and emerging markets as well as a cutting-edge methodology designed for analyzing the connectedness of markets.

In the tenth paper [10], the authors highlight the role of theoretical assumptions of the methods employed in the literature of energy markets. They show that the mathematical definition of chaos and the theoretical background are able to avoid possible errors from misleading results on the ostensible chaoticity of the price series. The findings indicate that both chaotic and stochastic features coexist in the energy commodity markets, although the misuse of some tests in the established practice in the literature may say otherwise.

The eleventh paper [11] focuses on the Mean Logarithmic Deviation, the measure proposed by Theil and based on the techniques of statistical information theory. The study investigates the role of age and education as the determinants of income inequality in Poland. The results confirm an association between the level of education and the average income of the groups distinguished on this basis. The study also finds that differences in the age of the household head had a smaller effect on income inequality than the level of education.

The twelfth paper [12] discusses the topic of uncovering causal interdependencies from observational data with the help of an information-theoretic concept known as the Rényi’s information measure. The authors investigate the directional information flow between bivariate time series in terms of the Rényi’s transfer entropy. The evidence is that the Rényi’s transfer entropy not only allows us to detect a threshold of synchronization, but it also provides non-trivial insight into the structure of a transient regime that exists between the region of chaotic correlations and synchronization threshold.

Finally, in the last paper of this Special Issue [13], the authors assess and compare changes in regularity in the 36 European and the United States stock market indices within major turbulence periods. Two periods are investigated: the Global Financial Crisis in 2007–2009 and the COVID-19 pandemic outbreak in 2020–2021. To capture sequential regularity in daily financial time series, the Sample Entropy algorithm is used. The empirical findings are unambiguous and confirm that the entropy of market indices decreases during turbulence periods, which implies that the regularity and predictability of stock market returns increases in such cases.

The high-quality contributions presented in this Special Issue offer a diverse and representative portfolio of entropy-based applications in economics, finance, and management. A wide variety of tools based on entropy confirms that entropy is presumably one of the most intricate scientific concepts. However, its comprehension is a challenge to researchers. As a guest editor, I hope that the readers will enjoy the papers included in this Special Issue and will find them interesting and helpful.
